# Digital Genotyping of Macrosatellites and Multicopy Genes Reveals Novel Biological Functions Associated with Copy Number Variation of Large Tandem Repeats

**DOI:** 10.1371/journal.pgen.1004418

**Published:** 2014-06-19

**Authors:** Manisha Brahmachary, Audrey Guilmatre, Javier Quilez, Dan Hasson, Christelle Borel, Peter Warburton, Andrew J. Sharp

**Affiliations:** Department of Genetics and Genomic Sciences, Icahn School of Medicine at Mount Sinai, New York, New York, United States of America; University of Minnesota, United States of America

## Abstract

Tandem repeats are common in eukaryotic genomes, but due to difficulties in assaying them remain poorly studied. Here, we demonstrate the utility of Nanostring technology as a targeted approach to perform accurate measurement of tandem repeats even at extremely high copy number, and apply this technology to genotype 165 HapMap samples from three different populations and five species of non-human primates. We observed extreme variability in copy number of tandemly repeated genes, with many loci showing 5–10 fold variation in copy number among humans. Many of these loci show hallmarks of genome assembly errors, and the true copy number of many large tandem repeats is significantly under-represented even in the high quality ‘finished’ human reference assembly. Importantly, we demonstrate that most large tandem repeat variations are not tagged by nearby SNPs, and are therefore essentially invisible to SNP-based GWAS approaches. Using association analysis we identify many *cis* correlations of large tandem repeat variants with nearby gene expression and DNA methylation levels, indicating that variations of tandem repeat length are associated with functional effects on the local genomic environment. This includes an example where expansion of a macrosatellite repeat is associated with increased DNA methylation and suppression of nearby gene expression, suggesting a mechanism termed “repeat induced gene silencing”, which has previously been observed only in transgenic organisms. We also observed multiple signatures consistent with altered selective pressures at tandemly repeated loci, suggesting important biological functions. Our studies show that tandemly repeated loci represent a highly variable fraction of the genome that have been systematically ignored by most previous studies, copy number variation of which can exert functionally significant effects. We suggest that future studies of tandem repeat loci will lead to many novel insights into their role in modulating both genomic and phenotypic diversity.

## Author Summary

Here we utilize Nanostring digital assays and show their utility for estimating copy number of 186 multicopy genes and tandem repeats. By analyzing patterns of single nucleotide variation around these variants, we show that copy number variation at the vast majority of tandem repeat variations is not effectively tagged by nearby SNPs, and thus standard genome-wide association studies that focus on SNPs provide little or no information about such variants. By comparing patterns of tandem repeat copy number with variation in local gene expression and DNA methylation, we also identify extensive functional effects on local genome function. This includes an example of a non-coding macrosatellite repeat, expansion of which exerts a repressive effect on a nearby gene accompanied by accumulations of local DNA methylation. Finally, comparison of diverse human populations with a number of primate genomes shows that many of these sequences have undergone extreme changes in copy number during recent human and primate evolution, and show signatures that suggest possible selective effects. Overall, we conclude that multicopy genes and macrosatellites represent a highly variable fraction of the genome with important functional effects that has been systematically ignored by previous studies.

## Introduction

More than half of the human genome is composed of various types of repetitive elements [1]. This includes tandem repeats, defined as stretches of DNA comprising two or more contiguous copies of a sequence of nucleotides arranged in head-to-tail pattern, for example CAG-CAG-CAG-CAG-CAG. Many tandem repeats are composed of repetitions of a short motif 1–6 bp, termed microsatellites, and these can often be highly polymorphic in copy number. However, some tandem repeats can be very large, with unit sizes of tens or even hundreds of kilobases [2]. Macrosatellites are defined as repeats with unit sizes ≥100 bp (also known as Variable Number of Tandem Repeats or VNTRs), and in some cases these large tandem repeats can include functional elements such as exons or entire genes within each repeat unit, resulting in potentially polymorphic multicopy genes.

Despite representing a significant source of genomic variation [3], tandem repeats were once labeled as mere ‘junk DNA’ and as a class of sequence variation remain very poorly studied. In part this has been due to the inherent technical difficulties in characterizing non-unique portions of the genome. For example, repetitive regions are typically excluded from microarray designs due to their high-copy nature. Even where they are targeted, techniques such as array comparative genomic hybridization (array CGH) typically do not perform well in repeats, as their high copy nature means that array signals often saturate at higher copy numbers [4]. Unless specialized approaches are used [5]–[7], even whole genome sequencing approaches tend to ignore tandem repeats due to the difficulty of mapping and interpreting reads in non-unique and highly variable parts of the genome. Finally, due to their multi-allelic nature and high mutation rate [8], most tandem repeat variants are thought to be poorly tagged by nearby SNPs [9]. Recent estimates derived from studies of >1 million transmissions in large Icelandic pedigrees have shown the mutation rate for microsatellites to vary between 2.5–3.5×10^−4^ for dinucleotide repeats, and 8.5–15×10^−4^ for tetranucleotide repeats [10]. Similar estimates for the mutation rate of SNPs suggest between 1.2–2.3×10^−8^ *de novo* mutations per site per meiosis [11], [12], several orders of magnitude lower than that of tandem repeats. As a result, it is likely that genome-wide association studies (GWAS) that typically rely on SNP genotyping approaches have yielded very little information for tandem repeat polymorphisms. Thus, tandem repeats represent a highly variable fraction of the genome that has been largely ignored by most high-throughput genomic technologies to date.

While microsatellite repeats have found common usage in linkage studies and forensics, several human disorders are known to be caused by extreme expansions of tandem repeats located within or near protein-coding genes [13]. This includes progressive myoclonus epilepsy involving CCCCGCCCCGCG expansions upstream *CSTB* [OMIM# 601145], Huntington disease caused by a CAG expansion within the coding region of *HTT* [OMIM# 143100], and Fragile X syndrome caused by a CGG expansion in the 5′UTR of *FMR1* [OMIM# 300624]. In addition to causing disease, changes in the length of microsatellites can exert effects on gene function and quantitative traits [14]–[16], and transcriptional plasticity [17], [18].

In contrast to microsatellites, macrosatellites and multicopy genes are less well studied. Although the functions of most multicopy genes located in tandem arrays are poorly characterized, recent studies have begun to associate copy number variation (CNV) of several of these with human traits. For example, CNV of the antimicrobial *β-defensin* genes at 8q23 is associated with susceptibility to psoriasis [19], [20] and dynamics of HIV infection [21], increased copy number of C4 protects against systemic lupus erythematosus [22], while copy number of tandemly repeated salivary amylase genes among different human populations is correlated with dietary starch consumption [23]. In these cases, alterations in gene copy number apparently operate directly on phenotype via a proportionate change in the resulting mRNA and protein levels. However, due to technical problems in reliably genotyping high copy number sequences, there is significant controversy regarding the contribution of multicopy genes to common disease susceptibility [24]–[34]. The conservation of some non-coding macrosatellites among primates, such as the DXZ4 repeat in Xq23, is suggestive of biological function [35]. When combined with their extreme variability and high mutation rate, this has led to the notion that CNV of tandemly repeated loci represents a rich reservoir of genomic variation that allows for rapid adaptive evolution in response to environmental change [36]–[38].

In some cases, CNV of tandem repeat arrays has also been associated with epigenetic effects. For example, the promoter expansions of the CGG repeat that causes Fragile X are associated with silencing of *FMR1* via hypermethylation [39]. On a larger scale, contractions of the D4Z4 macrosatellite that contains the *DUX4* gene, in combination with a permissive haplotype background, underlie facioscapulohumeral muscular dystrophy (FSHD) [reviewed in 40]. Reduced D4Z4 copy number is associated with a loss of DNA local methylation and heterochromatic histone marks [41], and is accompanied by an upregulation of local gene expression, although the mechanism causing this is unclear [42]. A similar phenomenon termed repeat induced gene silencing has been observed in several transgenic organisms [43]–[45]. Repeat induced gene silencing was first observed when transgenes that were integrated into a target genome in large tandem arrays were expressed at very low levels, despite being present at high copy number. In contrast, a reduction of transgene number to just a few copies paradoxically often resulted in high transgene expression. Repeat induced gene silencing apparently operates via an epigenetic mechanism in which repeat sequences are converted to a heterochromatic state [46].

In this study we have adapted Nanostring technology, a multiplexed digital counting method based on fluorescent barcodes initially developed for gene expression analysis [47], to study copy number variation of 173 multicopy genes, 2 intragenic coding repeats and 13 non-coding Macrosatellites. Here, we demonstrate the utility of Nanostring assays as a targeted approach that allows accurate tandem repeat genotyping even at extremely high copy number, and apply this technology to genotype 165 HapMap samples and five species of non-human primates. These data provide many novel biological insights into tandem repeat variations, including patterns of variation and linkage disequilibrium among different populations and evidence of selection during recent evolution. Our studies also identify strong correlations of macrosatellite copy number with local epigenetic marks and to a lesser extent with nearby gene expression, providing new evidence suggesting that repeat induced gene silencing might operate as a natural mechanism of gene regulation in humans.

## Results

### Probe Design and Validation of Data Quality

In order to identify multicopy genes with potential tandem architecture we utilized the “Join two Datasets” command in Galaxy (https://usegalaxy.org/), using the complete list of RefSeq genes as the two input files, with gene name as the joined field. We filtered the output to only retain genes that had >1 copy at a non-overlapping position on the same chromosome or chr_random, identifying 180 putative multicopy genes in the hg18 reference assembly. We designed a custom Nanostring probe set targeting 173 of these genes, in addition to 13 non-coding macrosatellites and 2 intragenic coding repeats (Table S1), and used this to genotype copy number in 165 HapMap individuals derived from three different ancestries (European, African and Asian) and five species of primates (Materials and Methods, Table S2). Based on manual curation of the genome assembly, we estimate that 102 of the 188 loci assayed (54%) show a clear tandem structure, 28/182 (15%) show a dispersed architecture where the multiple copies are interspersed with unique sequence, while the remaining 31% of sites have a genomic organization that is unclear or is not well assembled (see Materials and Methods).

We observed a strong association between features of poor genome assembly and these targeted loci. 33% (62 of 188) of our probes have one or more BLAT alignments of ≥95% identity on a chr_random, and 34% (60 of 175 genic probes) lie within 50 kb of an assembly gap, representing a >27-fold enrichment compared with all RefSeq genes. In total, 86 of the 337 (26%) euchromatic assembly gaps in the hg18 assembly lay within 50 kb of a multicopy gene, suggesting that the highly repetitive and polymorphic nature of multicopy genes are a significant source of genome assembly errors. As a result, the true copy number of such regions is often highly under-represented in the genome assembly. While the mean number of BLAT alignments of our probes in the haploid hg18 was 5.4 (median = 3), the estimated mean diploid copy number of these loci in the 165 HapMap individuals was 22 (median = 21.5).

**Figure 1:**
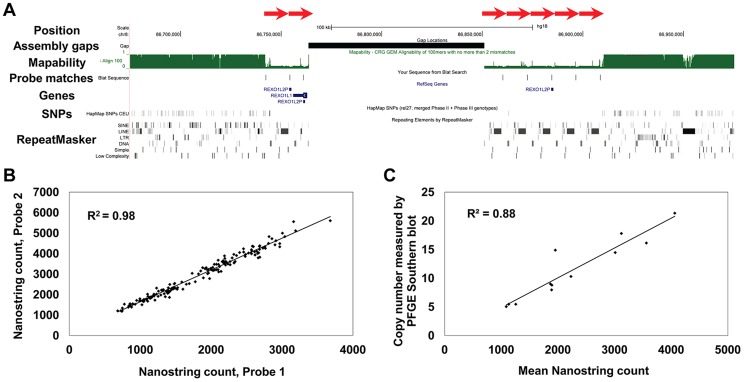
Structure and measurement of tandemly repeated genes using Nanostring assays. (**a**) Multiple copies of the *REXO1L1* gene occur as a tandemly repeated cluster in 8q21.2. Although just 4 copies of *REXO1L1* are annotated in hg18, at least seven copies of a ∼12.2 kb repeat are visible separated by a genome assembly gap (*red arrows*). Our studies show that this locus in fact varies from ∼110 to ∼250 diploid copies in normal humans. BLAT alignments show one of the two probes used to assay this locus that has a match to each of the annotated repeat copies, in addition to an unassembled copy on chr8_random (Table S1). Note the reduced mapability and almost complete absence of SNPs within the *REXO1L1* locus. Screenshot shows a 300 kb region of hg18 (chr8:86,675,000–86,975,000). (**b**) *CT47* is another gene present as a tandemly repeated cluster in Xq24. Measurement of *CT47* copy number using two independent probes targeted to different parts of the gene show extremely high concordance (R^2^ = 0.98), indicating that Nanostring probe counts provide accurate measurements that are directly proportional to copy number over a wide dynamic range. (**c**) Direct copy number estimation for *CT47* measured by Pulse Field Gel Electrophoresis (PFGE) in 12 individuals shows high concordance with Nanostring probe counts (R^2^ = 0.88).

To assess the reproducibility of Nanostring assays we first compared data generated from two independent probes, each of which target different parts of five genes that show highly variable copy numbers (*CT47*, *TBC1D3*, *PRR20A*, *REXO1L1*, and *CCL3/CCL4*). In each case, these independent replicates yielded highly concordant results (mean R^2^ = 0.88) (Figure 1, Figure S1), indicating that our measurements of DNA copy number are accurate and reproducible over a wide dynamic range. To further benchmark our data quality we compared copy numbers generated using Nanostring technology against existing ‘gold standard’ technologies for measuring high-copy number sequences. Copy number estimates based on direct fragment sizing of both the *CT47* tandem array by pulsed-field gel electrophoresis Southern blots [48] (Figure 1c) and *REXO1L1* [2], (Figure S2) showed excellent correlations with Nanostring counts (mean R^2^ = 0.92). We also compared Nanostring counts for the 8p23.1 *β-defensin* gene cluster with copy number measurements for this locus made using the paralog ratio test (PRT) [49], a method previously shown to be more accurate than qPCR for measuring high copy sequences [50]. Estimated copy numbers from both methods were highly correlated (R^2^ = 0.90, Figure S2). Thus, Nanostring assays provide copy number estimates whose accuracy equals or exceeds that yielded by other current targeted genotyping technologies.

### Analysis of Copy Number Variation in Three Human Populations

While Nanostring assays provide counts that are proportional to underlying copy number, relative counts given by different assays can vary depending on individual probe efficiencies. To derive absolute copy numbers in each individual, we therefore compared Nanostring probe counts with corresponding copy number estimates from whole-genome shotgun read depth analysis from the 1000 Genomes Project [51]. Based on individuals who were tested by both Nanostring and read depth analysis, for each probe we converted Nanostring counts to absolute copy number in each individual using the median read depth and Nanostring counts for each locus as calibration points (see Materials and Methods). Scatter plots showing correlations between each Nanostring probe and the estimated copy number by read depth analysis are shown in Figure S3. Overall these data showed that the performance of Nanostring assays appears comparable to that obtained by read depth analysis.

116 of the 186 (62%) loci tested showed copy number variation among the 165 HapMap individuals. Several of these loci (*DUX4*, *NBPF10*, *USP17* and *REXO1L1*) showed mean diploid copy numbers >100 within the normal human population, and these loci also showed the highest variability (Table S3). For example, total copy number of *DUX4*, which occurs in two large tandem clusters on chromosomes 4 and 10, ranges from 200 to 685 in HapMap individuals. Similarly, 5 of the 13 macrosatellites tested showed mean copy numbers >100. In contrast, the mean copy number of these five macrosatellites Sats in the hg18 reference assembly is 26, indicating that the genome assembly at many of these tandemly repeated loci is collapsed, and systematically under-estimates the true copy number of these repetitive regions.

**Figure 2:**
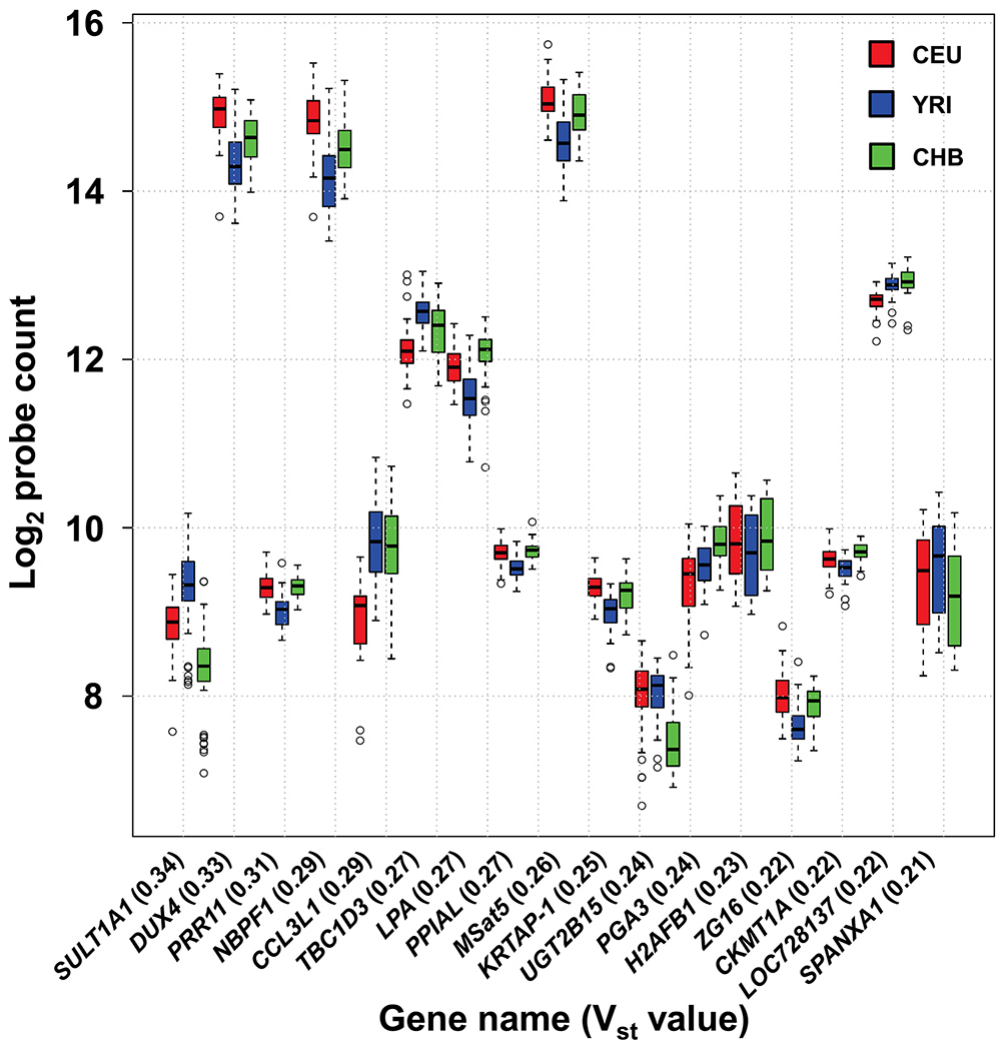
High frequency of population stratification for CNV of multicopy genes. 17 of 116 (14.7%) multicopy genes show high levels of differentiation in copy number (V_st_>0.2) among European, African and Asian populations. Note that probe counts on the y-axis are shown on a log2 scale.

We also observed highly differentiated copy numbers across the three different HapMap populations sampled (60 CEU, 60 YRI and 45 CHB). To quantify this inter-population variance we calculated ANOVA-F_ST_ (V_ST_) values for each probe in our design. Elevated (>0.2) values of V_ST_ indicate sites with highly divergent copy numbers in different populations, which can be a signature of positive selection [52]. One macrosatellite (MSat5) and 16 multicopy genes showed V_ST_ values >0.2 among European, African and Asian populations (Figure 2), indicating significant population differentiation at these loci. The most highly differentiated locus identified, *SULT1A1*, a gene involved in detoxification of environmental chemicals such as catecholamines and phenolics, showed a mean copy number of 5.7 in Europeans, 4.8 in Chinese, and 7.7 in Africans. These data are consistent with previous studies that have identified population differences in SULT1A1 enzyme activity between Africans and Europeans that is linked with copy number [53].

We performed an intersection of our probe locations with a list of 8,598 CNVs detected by a previous array CGH study that utilized ∼21 million probes spaced throughout the human genome [52]. After excluding duplicates and probes removed due to poor performance, 97 of 186 loci tested by our Nanostring assay (52%) overlapped with CNVs defined by Conrad et al. (Table S3). Of the 89 loci that were not reported as CNV by Conrad et al., 37 showed evidence of CNV in the HapMap population using our Nanostring assays, demonstrating that many of the loci we targeted have been ignored by even the most thorough studies of CNV.

### Linkage Disequilibrium Analysis of Multicopy Genes and Macrosatellites

**Figure 3:**
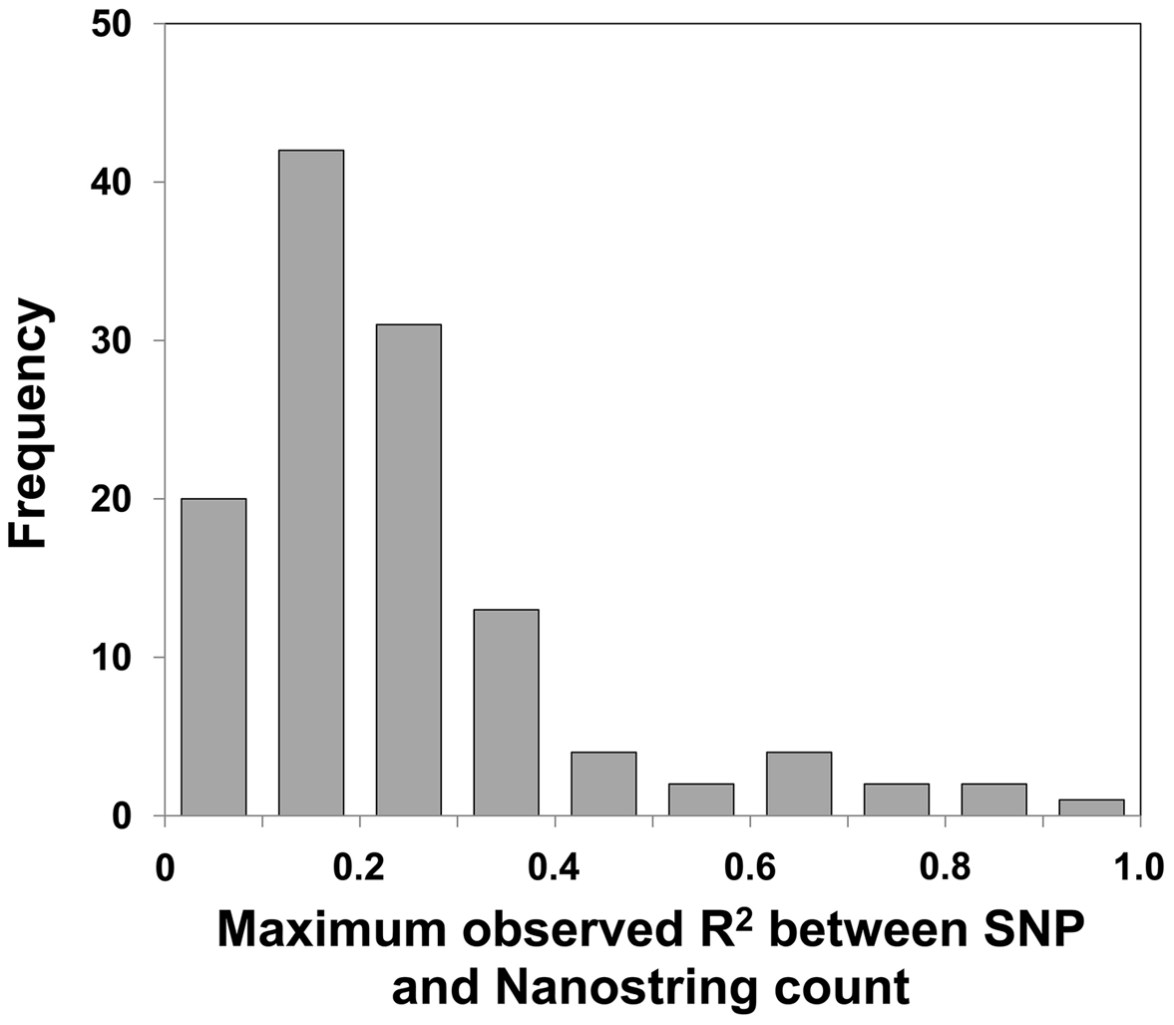
Most multicopy genes show very low levels of linkage disequilibrium with nearby SNPs. Correlation analysis for each of the 121 polymorphic probes targeting multicopy genes and macrosatellites with SNP markers within ±250 kb yielded a median R^2^ = 0.18 between the highest ranked filtered SNP and probe count. Only 3 of 116 (∼3%) multicopy genes showed an R^2^≥0.8 with any SNP in the three populations studied. Therefore the vast majority of tandem repeat variations lack informative tag SNPs, and thus association studies of multicopy loci require specific genotyping of each locus to gain accurate copy number information of these regions.

Previous studies of simple bi-allelic insertion/deletion CNVs in HapMap samples have shown that 77% of these CNVs have one or more tagging SNPs with R^2^>0.8 [52]. In order to assess the ability of SNP-based approaches such as GWAS to interrogate more complex CNVs such as multicopy genes and macrosatellites which can have multiple different allelic states, we measured the relationship between CNV of these regions and nearby SNPs. Firstly we observed a 4.5-fold reduced SNP density in these regions versus the genome average. Based on CEU HapMap Phase II data, regions within ±25 kb of each probe alignment showed a mean density of one SNP per 3.3 kb, versus a genome mean of one SNP per 738 bp. We searched for SNPs that effectively tag copy number within ±250 kb of each locus assayed (Tables S4 and S5). Even when considering the best filtered tag SNP identified in any of the three HapMap populations (CEU, YRI, CHB, or all combined), analysis of the 116 variable loci yielded a median R^2^ = 0.22 between the top ranked SNP and probe count. Only four loci (3.4% of the loci tested) showed an R^2^≥0.8 with any SNP in at least one of the three populations studied (Figure 3). Thus, CNV of the vast majority of multicopy genes and macrosatellites we analyzed are not effectively tagged by flanking SNP markers. Similar results were obtained after excluding loci with a dispersed architecture (see Materials and Methods), total copy number of which would be expected to be poorly tagged by any one SNP marker (data not shown).

### Influence of Copy Number Variation on Local Gene Expression

To measure the functional effects of changes in copy number of tandemly repeated sequences we performed correlations with local gene expression levels in human lymphoblastoid cell lines. We used published gene expression data for 58 CEU and 59 YRI HapMap individuals [54], and tested genes ±500 kb of each probe alignment in both CEU and YRI populations separately, and as a single combined group. Overall, we identified 138 genes whose expression level showed significant correlation with probe counts of the 116 variable loci (permutation p<0.01, Table S6). The strongest correlation was observed at *GSTM1*, for which 77% of the variance in gene expression was attributable to underlying gene copy number (Table S6). Somewhat surprisingly, in most cases the transcripts whose expression correlated with copy number were located outside the region of known CNV (median separation between probe and gene transcription start site 208 kb). We observed only eight instances (∼6% of the observed expression correlations) in which copy number of the gene being interrogated was auto-correlated with its own expression level in lymphoblastoid cells (*FCGR2A*, *GSTM1*, *GSTT1*, *KRTAP1-3*, *LCE2D*, *MAGEA11*, *TRGV5* and *UGT2B15*). We obtained very similar results when using published gene expression data generated using RNAseq (data not shown) [55], [56].

### Influence of Copy Number Variation on Local DNA Methylation

**Figure 4:**
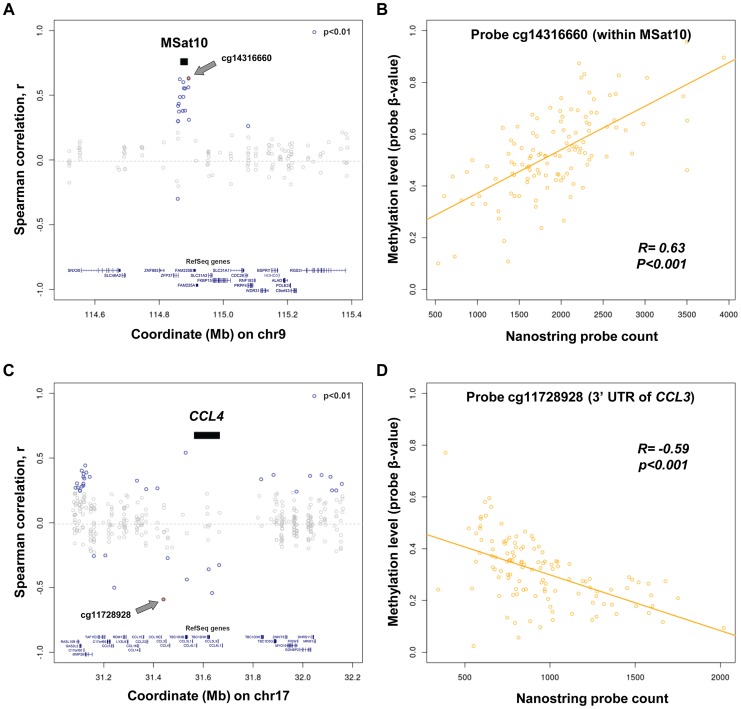
Variation in copy number of tandem repeats and multicopy genes is associated with alterations of local DNA methylation. (a and c) Shown are correlation values between copy number of (a) MSat10 and (c) *CCL4* with all methylation probes within ±500 kb in 118 CEU and YRI HapMap individuals. (b and d) Scatter plots showing individual level data for the methylation probes showing the strongest correlations with copy number of Msat10 and *CCL4*. (b) Increasing copy number of MSat10 is associated with increased methylation levels of cg14316660, (R = 0.63, permutation p<0.001). This association was replicated using a Sequenom assay targeted to the MSat10 locus (Figure 5c), confirming that it is not simply due to a technical artifact related to CNV of the underlying probe binding sites. (d) Increasing copy number of *CCL4* is associated with reduced methylation levels of cg11728928 (R = −0.59, permutation p<0.001). In (a) and (c), black bars indicate the interval to which each Nanostring probe maps, CpGs showing correlation p<0.01 are indicated in blue, while the CpG showing the strongest correlation is shown as a filled blue circle and labeled with a grey arrow (with individual data plotted in (b) and (d), respectively).

To further assess functional effects of changes in copy number of tandemly repeated sequences we performed correlations with local DNA methylation levels in human lymphoblastoid cell lines. We used published CpG methylation data for 60 CEU and 58 YRI HapMap individuals [57], and tested CpGs within ±500 kb of each probe alignment in both CEU and YRI populations separately, and as a single combined group. Overall, this analysis identified 5,147 individual CNV:methylation pairwise correlations that were observed in either CEU, YRI or both populations combined (p<0.01, Table S7). Focusing only on those correlations that were associated with large changes in methylation (absolute normalized slope >0.1) and which were observed in each of CEU, YRI and the combined populations independently identified 65 autosomal and 25 X-linked CpGs whose methylation levels showed consistent strong correlations with nearby multicopy loci (permutation p<0.01, Tables S8 and S9). Some of the strongest associations were observed at the MSat10 locus, CNV of which showed positive correlations with multiple CpGs spread over ∼50 kb including the MSat10 tandem repeat itself and the promoter of the adjacent *ZFP37* gene. In contrast, the strongest negative correlations were observed for multiple CpGs around the *CCL3*/*CCL4* locus (Figure 4).

### Indications of Repeat Induced Gene Silencing Associated with Macrosatellite Repeats

**Figure 5:**
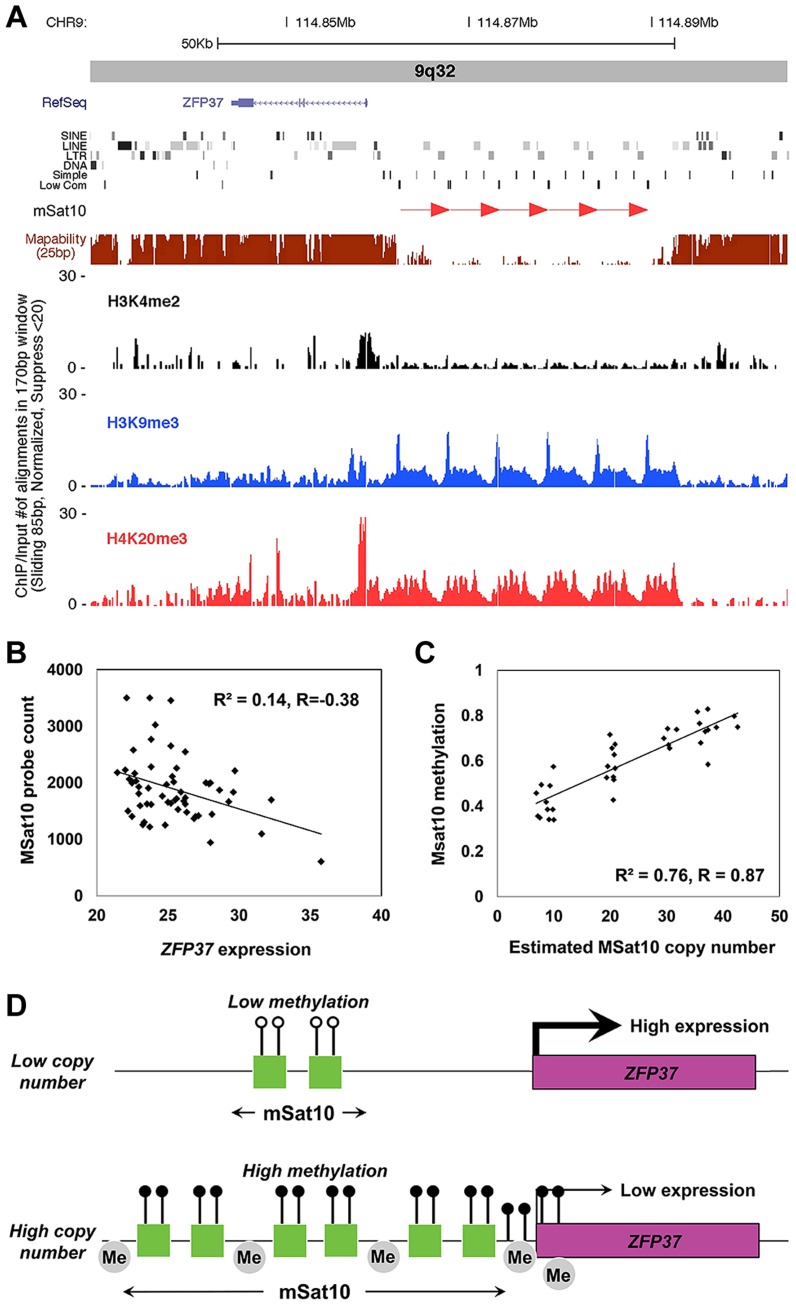
Association of MSat10 copy number with neighboring gene expression and epigenetic marks. (**a**) MSat10 is a 5.2 kb GC-rich tandem repeat that lies ∼4 kb distal to the gene *ZFP37*. Although 6 copies of this 5.2 kb repeat are present in the hg18 assembly this macrosatellite is highly polymorphic in size, varying from 4–42 copies in HapMap. ChIP-seq analysis shows the presence of histone marks characteristic of heterochromatin, such as trimethylation of histone H3 at lysine 9 and trimethylation of histone H4 at lysine 9. Screenshot from the UCSC Genome Browser shows *ZFP37* (Zinc Finger Protein 37), the adjacent MSat10 repeat (*red arrows*), and the results of ChIP-seq analysis. (**b**) In 58 unrelated CEU HapMap individuals we observed an inverse correlation between copy number of the MSat10 repeat and expression level of the adjacent gene *ZFP37*, demonstrating suppression of *ZFP37* expression associated with larger repeat sizes (**c**) Using a targeted Sequenom assay, we confirm that variable methylation of MSat10 is highly correlated with repeat number (R^2^ = 0.76, p = 4.4×10^−12^), showing a strong relationship between repeat size and local epigenetic state. (**d**) Proposed model of repeat induced gene silencing at the MSat10 locus. At low repeat numbers the region is euchromatic and the expression of the neighboring *ZFP37* gene is high. However, expansions of the macrosatellite result in an accumulation of heterochromatic marks in the region, including repressive histone modifications and DNA methylation, resulting in the suppression of local gene expression. Although our model shows methylation on all MSat10 copies, our data does not exclude the possibility that on expanded MSat10 alleles DNA methylation is limited to a subset of the repeat units. Lollipops represent DNA methylation, with open circles being low and filled black circles high DNA methylation, and grey ‘Me’ bubbles represent repressive histone methylation.

Using gene expression data from lymphoblastoid cell lines, we observed two cases where the expression level of a gene lying adjacent to a macrosatellite was inversely correlated with repeat copy number, suggesting a mechanism in which expansion of these repeats suppresses local transcription. Expression of *ZFP37* showed a weak but significant inverse correlation with the copy number of MSat10 (R = −0.38, p = 0.0042), a 5.4 kb a GC-rich repeat (60% G+C) which lies 2 kb proximal and varies between 4–43 diploid copies in human (Figure 5). Similarly expression of *ZNF558* was inversely correlated (R = −0.46, p<0.0001) with copy number of the adjacent MSat12, which lies 24 kb distal. This correlation of MSat12 copy number with *ZNF558* expression was detected independently in both European and African populations, showing that these effects are reproducible and not specific to single ethnic groups (Table S6).

Based on these observations, we hypothesized that this suppression of gene expression at high macrosatellite copy numbers might represent an epigenetic mechanism known as repeat induced gene silencing in which the chromatin environment around large tandemly repeated arrays become heterochromatic, leading to local transcriptional repression [44], [58], [59]. Consistent with this hypothesis, we noted that one of the strongest correlations we detected between tandem repeat copy number and DNA methylation also occurred between MSat10 and CpGs within the local region, including those at the promoter of *ZFP37* (Figure 4, Table S8). This observation was confirmed using a Sequenom MassARRAY EpiTYPER assay in 40 HapMap individuals of diverse ancestry, which showed highly variable DNA methylation at MSat10 that showed a strong positive correlation with repeat copy number (R^2^ = 0.76, p = 4.4×10^−12^, Figure 5c). Chip-seq analysis also showed high levels of repressive histone H3 trimethylation of lysines 9 and histone H4 trimethylation of lysine 20, and an absence of activating H3 dimethylation of lysine 4 at the MSat10 locus, with spreading to *ZFP37*. Analyses at MSat12, MSat6, MSat9 and MSat14 showed no significant variation of DNA methylation levels at these loci.

### Evolution of Multicopy Genes in Primate Species

**Figure 6:**
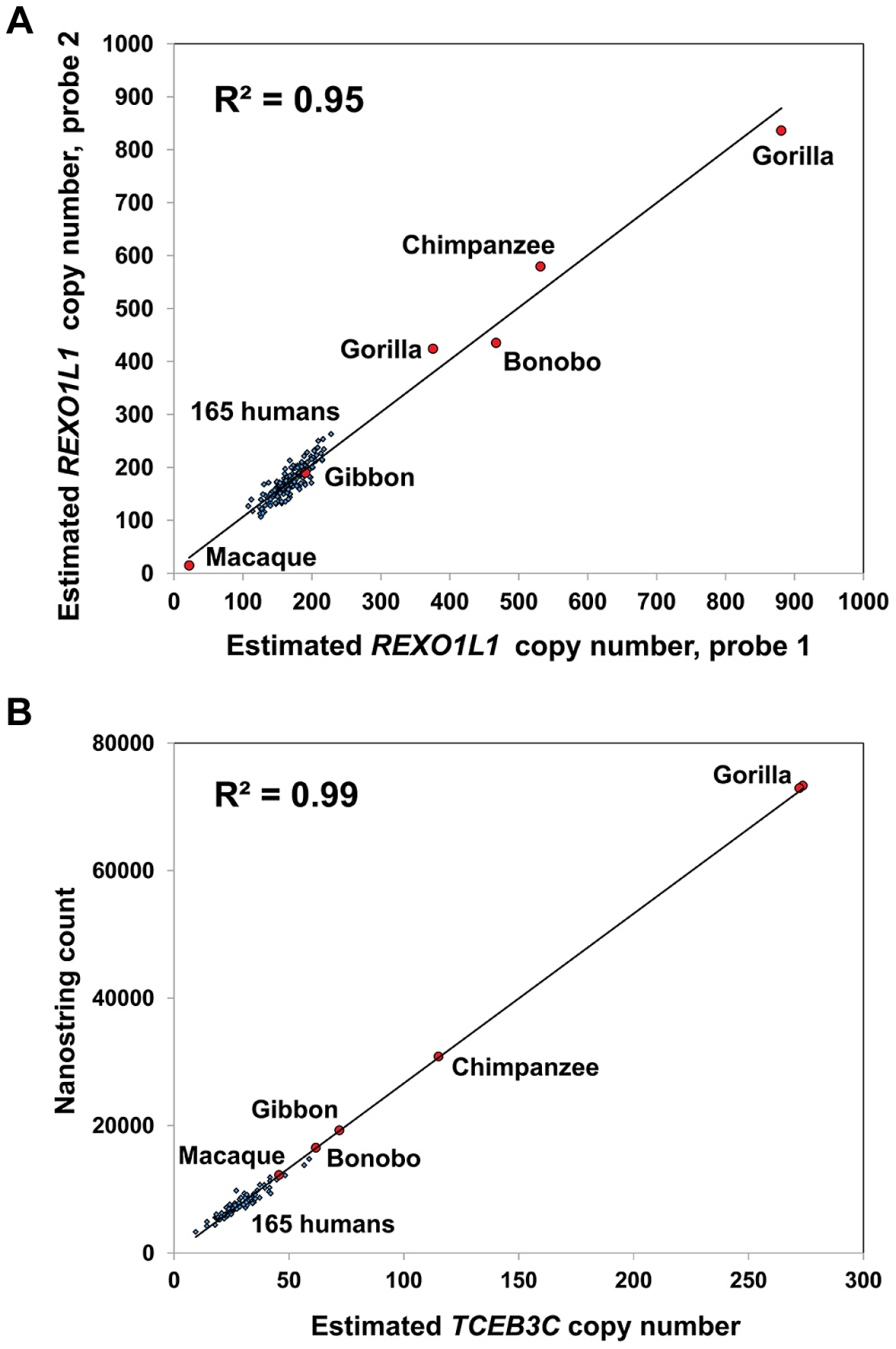
*REXO1L1* and *TCEB3C* show extreme variation in copy number among primate species. (**a**) *REXO1L1* is one of the most extreme examples of copy number variable genes in human, with 108–266 copies of the ∼12.2 kb repeat unit observed in the 165 HapMap individuals studied. However even more extreme variation is observed among different primates. We observed ∼450 and ∼550 copies in bonobo and chimpanzee, respectively, and copy numbers of ∼400 and ∼860 in two different gorilla individuals. In contrast while macaque has an estimated 22 copies, gibbon falls within the same range seen in human. (**b**) While *TCEB3C* ranges from 9–59 copies among HapMap individuals (mean 29 copies), all five species of primate studied show increased copy number, indicating a reduction of *TCEB3C* copy number specifically in the human lineage. As with *REXO1L1*, gorilla and chimpanzee showed the highest copy numbers, with 115 in chimpanzee and ∼270 copies in both gorillas studied.

To gain insight into copy number changes of tandem repeat loci during primate evolution we genotyped six DNA samples from five different primate species using our Nanostring assay: chimpanzee, bonobo, two gorilla individuals, gibbon, and macaque (Table S10). 20 multicopy genes and macrosatellites showed ≥3-fold difference in copy number in at least one primate species versus the mean copy number observed in humans (Figure S4), and several showed extreme changes among primates. For example, *REXO1L1* has a mean of 171 copies in human, but was present in ∼860 copies in one of the gorilla samples analyzed (Figure 6). Similarly *TCEB3C* showed significantly increased copy numbers in several primates, with 115 copies in chimpanzee and ∼270 in gorilla, compared to a mean of 29 copies in human, while *PRAMEF14* shows a mean of 11 copies in human, and ∼200 in chimpanzee. Contrastingly, we also observed genes such as *GAGE6* that show apparent human specific expansion, with a mean of 40 copies in HapMap individuals compared to much lower copy number in all primate species tested.

**Figure 7:**
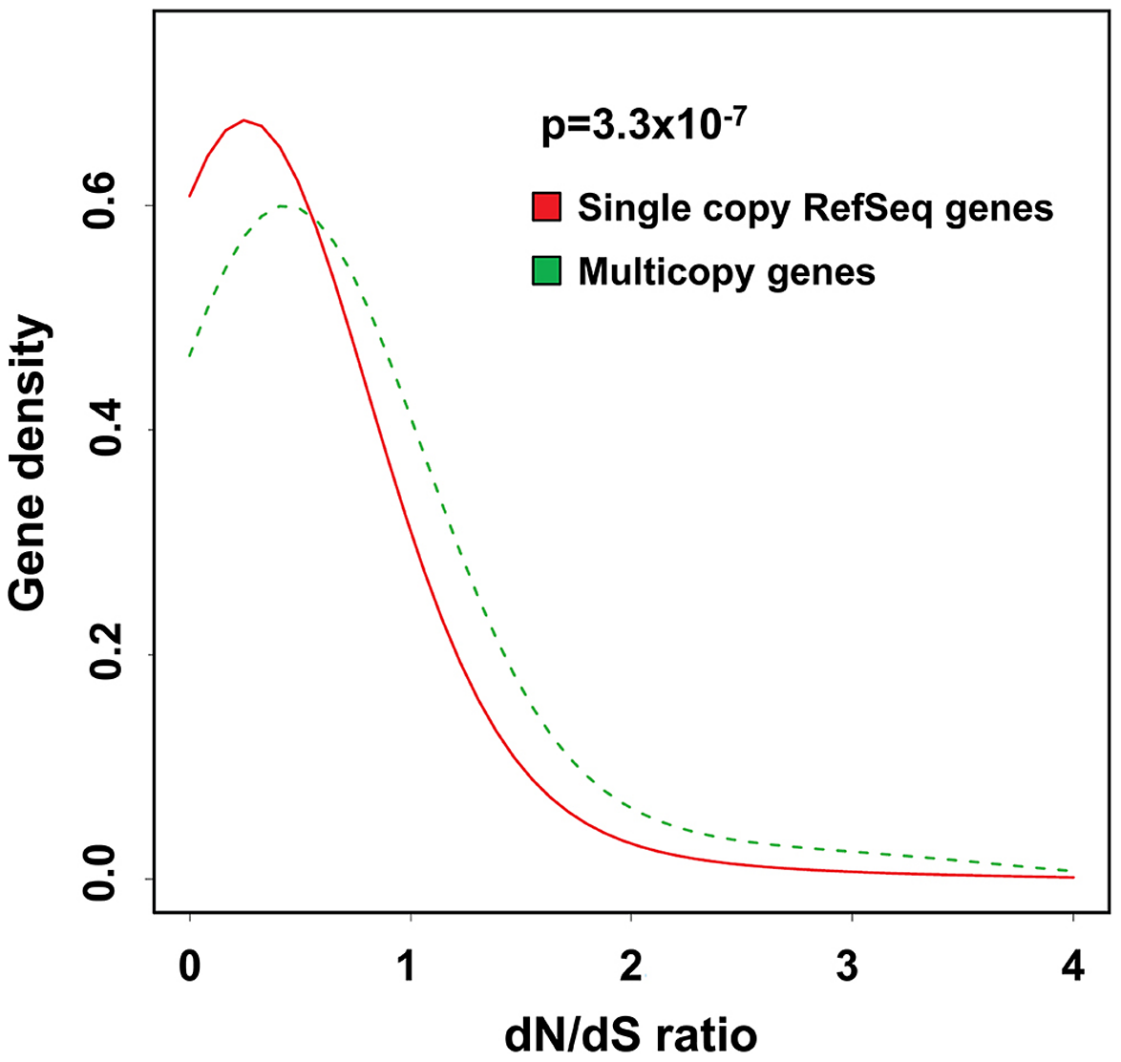
Multicopy genes show evidence of altered selective pressures on amino acid sequence during recent primate evolution. Density plots showing the distribution of dN/dS ratios for multicopy genes (*green*) compared to all RefSeq genes (*red*) for human versus chimpanzee. There is a significant enrichment for elevated rates of non-synonymous substitution in multicopy genes versus the genome average (p = 3.3×10^−7^, Kolmogorov-Smirnov test). This excess of non-synonymous amino-acid changes in recent primate evolution at multicopy genes is consistent with either reduced selective constraint and/or selection for proteins with altered function. Similar results are obtained when comparing human with orangutan and macaque (Figure S5).

Given the high diversity of copy number for many multicopy genes, both among different human populations and between different species of primate, we characterized the rate of synonymous (dS) to non-synonymous (dN) amino-acid replacement between humans and other primates (chimpanzee, orangutan and macaque) as an additional measure of evolutionary selection. Using a curated set of human:primate orthologs [60], we utilized the reference genome sequences for human, chimpanzee, orangutan and macaque to calculate the number of synonymous and non-synonymous protein-coding variants between species within each gene. We observed a consistent and highly significant increase in the distribution of dN/dS ratios of multicopy genes versus the genome average, suggesting altered selective pressures on multicopy genes when compared to single-copy genes. When comparing the amino acid sequences of human and chimpanzee, 12 of 97 multicopy genes (12%) which were tested by our Nanostring assay showed dN/dS>1 compared to 1,108 of 16,261 (6.8%) single copy RefSeq genes (Figure 7). Similarly, comparing amino acid sequences of human and orangutan, 11 of 90 multicopy genes we tested (12%) showed dN/dS>1 compared to 645/16,532 (4%) unique genes, while in the human:macaque comparison, 10 of 89 multicopy genes (11%) had dN/dS>1 compared to 348/16,557 (2%) unique genes (Figure S5).

## Discussion

Utilizing a novel multiplexed digital genotyping technology, we have performed a detailed analysis of copy number variation of multicopy genes and tandemly repeated macrosatellites in the human genome. We identify many interesting biological insights at these loci. This includes (i) extremely high levels of CNV among both different human populations and primate species; (ii) an almost universal lack of LD between CNV of these regions and nearby SNP markers, meaning that CNV of the vast majority of multicopy genes and macrosatellites is not tagged by flanking SNP markers, and that effective interrogation of these loci requires specific methods capable of effectively assaying these regions; (iii) significant associations between tandem repeat copy number and local gene expression and epigenetics; and (iv) signatures suggestive of positive selection acting at many of these loci. Thus, despite being largely ignored by most genome-wide studies, this class of genomic variation shows many features that suggest important functional effects.

Of particular note, our analysis identifies an example consistent with repeat induced gene silencing operating in normal human genomes associated with increased copy number of MSat10, a GC-rich 5.4 kb tandemly repeated motif located in 9q32 (chr9:114,862,000–114,893,000). We observed a strong positive correlation between copy number of MSat10 and local DNA methylation level (R^2^ = 0.76), together with a weaker but significant inverse correlation between MSat10 copy number and expression of the adjacent gene *ZFP37*, which lies 2 kb proximal to the repeat. ChIP-seq analysis also showed the presence of heterochromatic marks associated with MSat10 which extend proximally to *ZFP37*. Thus our data suggest heterochromatinization of the MSat10 locus at high repeat copy number with a resulting suppression of local gene expression. A similar inverse relationship was also observed between MSat12 copy number in 19p13.2 and expression of the adjacent gene *ZNF558*, which lies 24 kb distal to the repeat, although we did not detect any significant inter-individual variation of DNA methylation at this locus. Based on transgenic studies, it has been proposed that repeat induced gene silencing represents a system for the protection of eukaryotic genomes against hostile sequence elements that can integrate into the genome in high copy numbers, such as viruses and transposons [59]. However, our study suggests that repeat induced gene silencing also operates as a natural mechanism of gene regulation associated with tandem repeat tracts in normal genomes. While this study has focused only on the very largest tandem repeats present within the human genome, the high prevalence of tandemly repeated sequences in mammalian genomes means that repeat induced gene silencing may represent a more common mechanism of genetic regulation than is currently appreciated. In particular, as we have utilized gene expression data obtained from a single cell type (lymphoblastoid cell lines), our conclusions are limited to those genes that are expressed in this system. This fact likely underlies the relatively small number of genes that we observe that show auto-correlations of copy number and gene expression level. For example, for all other macrosatellites we profiled apart from MSat10 and MSat12, the closest genes expressed at measurable levels in lymphoblastoid cells were located >100 kb from the repeat. In contrast, *ZFP37* and *ZNF558* lie just 2 kb and 24 kb from the macrosatellites whose copy number they are inversely correlated with, respectively.

Notably we observed that the true copy number of many tandem repeat arrays is significantly under-represented even in the high quality ‘finished’ human reference assembly. Most likely this is a result of mis-assembly artifacts which tend to result in the collapse of repeat sequences [61]. One of the most extreme cases is that of the *REXO1L1* tandem array. The hg18 assembly contains eight assembled copies of this gene in 8q21.2, in addition to an 87.3 kb assembly gap and a further two unassembled copies on chr8_random. In contrast, our studies show that the ∼12.2 kb repeat containing the *REXO1L1* gene has a mean of 173 diploid copies in HapMap individuals. Our data therefore indicates that the true mean length of each haploid *REXO1L1* tandem array is therefore on the order of ∼1 Mb. Consistent with this a previous analysis of two pedigrees by PFGE showed alleles ranging from ∼700 kb to ∼1.6 Mb in size [2], while the identification of individuals with cytogenetically visible expansions of 8q21.2 has shown that in some cases the *REXO1L1* array may be considerably larger [62]. Of note, our analysis of primate genomes shows that the *REXO1L1* tandem array has expanded to even greater copy numbers in chimpanzee, bonobo and gorilla, with ∼860 copies observed in one gorilla individual. Although the function of *REXO1L1* is unknown, previous studies suggest a link between its protein product GOR and hepatitis C infection and autoimmune liver disease [63]–[65].

Why do some genes exist in multicopy arrays? Recent data in plants suggest that expanded tandem gene arrays can modify phenotypes such as disease resistance via over-expression of genes within the repeated segment [66]. As such, we propose that genes present in highly variable tandem arrays represent strong candidates for variations in human phenotypes, including disease susceptibility. Our analysis demonstrates that the vast majority of such variants are not effectively tagged by nearby SNP markers, and thus CNV of multicopy genes and macrosatellites represents a class of genetic variation that is essentially invisible to conventional SNP-based GWAS. Such variants therefore represent an attractive candidate to account for a portion of the so-called ‘missing heritability’ of the genome [67], [68]. Indeed, increased copy number of antimicrobial *β-defensin* genes has already been implicated as a risk factor for both psoriasis [19], [20], and HIV load [21], associations which were only detected after targeted studies of *β-defensin* copy number were used. Although most are poorly studied, we note that many other genes present in multicopy arrays also have links with immune function, suggesting that CNV of these loci represents a potential candidate contributing towards the heritable risk of diseases with infectious or autoimmune components [22]. We suggest that association studies that focus specifically on CNV of multicopy genes as risk factors for human phenotypes will be a fruitful line of future research. While the use of read depth from whole-genome sequencing can provide accurate estimates of gene copy number [51], this approach is still expensive and the analysis time consuming. In contrast, Nanostring technology represents an attractive alternative for performing such studies, as it is a simple single tube assay that is capable of providing digital relative copy number estimates for dozens of loci at relatively low cost per sample, with a potential throughput of hundreds of samples per week. While Nanostring assays alone only give relative, and not absolute copy number counts, this information is not necessary for association studies, and inclusion of just a few samples of known copy number allows simple calibration to be performed.

Our analysis shows Nanostring assays represent a novel method that allows accurate estimation of high copy number sequences over a wide dynamic range, making this technology well suited for studies of high-copy sequences such as large tandem repeats. One limitation of this technology is that like many other methods it does not provide allelic copy number, instead simply giving a composite measure of total genomic copy number. As a result, our study likely under-estimates the true extent of allelic diversity at many tandemly repeated loci. Much more laborious methods such as PFGE [2], fiber FISH [23] molecular combing [69], or optical mapping approaches [70] are currently the only reliable methods of estimating allelic copy numbers. Another limitation is the lack of information on genomic location. While many multicopy genes occur as tandem arrays at a single genomic locus, others are dispersed at different genomic locations. This fact could explain the lack of LD with neighboring SNP markers observed for some loci. Finally assay design for Nanostring probes requires prior knowledge of the sequence(s) to be targeted, and as it is based on hybridization, is unable to distinguish between highly identical paralogs. Because of the targeted nature of our probe design and the inherent limitations of the genome assembly, it is difficult to accurately assess what fraction of large tandem repeat arrays present in the human genome we have assayed in this study. While we have probably captured the majority of assembled large tandem repeats, it is also clear that there are other macrosatellites that we did not assay, e.g. the RNU2 locus [69], and likely others that are not correctly represented in current genome assemblies. The future development of very long read sequencing technologies will be required in order to fully resolve the true structure of many tandemly repeated loci.

Our studies revealed multiple signatures that might indicate positive selection operating on multicopy genes. Firstly, among European, African and Asian populations we observed unusually high levels of divergence of copy number at many of the loci studied. Second, many multicopy genes also show highly divergent copy numbers among different primate species, with many loci showing >3-fold increases compared to the average in human. Of note, this in fact likely represents an under-estimate of the true human-primate divergence given the reduced hybridization efficiency of some probes that contain mismatches against different primate genomes. Third, we observed a significant excess of non-synonymous amino-acid changes in recent primate evolution at multicopy genes. Although when considered individually these signatures do not provide conclusive proof of altered selection, the fact that all three analyses yielded concordant results is suggestive of altered evolutionary pressures operating on both the copy number and protein sequence of many multicopy genes, which is consistent with either reduced selective constraint, or selection for proteins with altered functions. We note that previous studies of multicopy genes included in our analysis have also identified strong signatures of selection at several of these loci [23], [49], [71]–[73]. However, it should also be noted that measurements of amino acid divergence at many of the genes we included in our study could be confounded by the presence of multiple paralogs, and thus this requires future confirmation.

One further consequence of the presence of genes with multiple copies is that they readily provide a mechanism for acquiring divergent functions through the occurrence of subsequent coding or regulatory mutations in different copies [74]. For example, evolution of polychromatic color vision in primates occurred as a result of sequence divergence among different members of the tandemly arranged opsin gene family [75], [76], and structural variation of the opsin gene cluster underlies red-green color blindness [77], [78]. Thus, tandem gene arrays can both provide a substrate for the evolution of novel protein functions, and act as a reservoir of significant phenotypic variation within a population.

Although often ignored in genome-wide analyses, our data show that many large tandem repeats are extremely variable, and CNV even of non-coding tandem repeat loci can be associated with significant functional effects on the genome. We suggest that more detailed studies of tandem repeat loci will lead to many novel insights into their role in modulating both genomic and phenotypic diversity.

## Materials and Methods

### Selection of Multicopy Genes

180 multicopy genes were identified by performing a self-join operation using RefSeq to identify genes that had >1 copy at a non-overlapping position on the same chromosome or chr_random. Custom Nanostring probes were successfully designed for 173 of these genes, in addition to two intragenic tandem repeats (*LPA* and *SPDYE3*), and 13 non-coding macrosatellite repeats identified in a previous study [2]. Macrosatellites represented large tandem repeat regions, with repeat unit sizes varying from 1.8–7.7 kb, each annotated with multiple copies in the reference genome. For each locus included on our probe design we performed visual assessment in the UCSC Genome Browser, comparing to annotations such as segmental duplications, RepeatMasker and gene structures. Based on this assessment we classified the local architecture as either (i) tandem (the multiple copies showed a clear serial arrangement), (ii) dispersed (no clear evidence of tandem structure), or (iii) the true architecture is unknown, (e.g. genes that had one mapped copy and a second unassembled copy on the corresponding chr_random). In total, we estimate that 102 of the 188 loci assayed (54%) show a clear tandem structure, 28/182 (15%) show a dispersed architecture, while the remaining 31% of sites have a genomic organization that is unclear or is not well assembled. Thus, overall, while the majority of sites we assayed represent tandem repeat variations, a significant fraction has a dispersed architecture, with multiple copies separated by large segments of intervening sequence.

We designed a custom Nanostring probe set targeting these 186 multicopy genes and macrosatellites, and included additional probes for *SRY* and an invariant X chromosome locus as gender controls. Each locus was targeted by a single probe, except for five highly variable loci (*REXO1L1*, *PRR20A*, *TBC1D3*, *CCL3/CCL4* and *CT47*) for which we designed two independent probes targeting different parts of the gene to measure assay reproducibility. This probe set was used to genotype 165 unrelated Phase I HapMap individuals (60 CEU, 60 YRI, 45 CHB), and five species of primate (one *Pan troglodytes* (chimpanzee), one *Pan paniscus* (Bonobo), two *Gorilla gorilla* (gorilla) individuals, one *Hylobates sp.* (gibbon), and one *Macaca arctoides* (Stump-tailed macaque)) (Table S7).

Each probe sequence was queried against the hg18 human reference genome using BLAT. Alignments of ≥90% identity, allowing for 1 bp of insertion/deletion, are shown in Table S1. By nature, each probe had multiple BLAT alignments in the human genome, with 65 of 195 probes (33%) having matches of ≥95% identity to chr_random, and the number of BLAT alignments per probe ranging from 2 to 74 (median 3). Probe sequences were also queried against primate genomes of *Pan troglodytes* (panTro2), *Gorilla gorilla* (Gor3.61) and *Macaca mulatta* (rheMac2). BLAT results ≥90% identity, allowing for 2 bp of insertion/deletion, are shown in Table S10.

### Normalization and Background Correction

The probe set included eight negative control probes that target artificial sequences, and ten normalization probes that target autosomal loci that are invariant in copy number. Additional probes for *SRY* and invariant X chromosome loci were included as both gender controls and to provide internal performance control data between males and females. Two probes (*SCXB* and *TAF9B*) yielded mean counts <100 across all HapMap individuals and were excluded from further analysis. Background subtraction was first performed on raw counts by subtracting the mean count of the eight negative control probes in each sample from the raw count of all test probes. The ten invariant autosomal probes were then used to derive a normalization factor for each sample to account for technical variables such as varying amounts of input DNA or hybridization efficiency between assays. The normalization factor per sample was derived by calculating the mean count of the ten invariant control probes in that individual divided by the mean count for all normalization probes across all samples. Raw counts for each of the 195 probes in each individual where then multiplied by the normalization factor for that sample to produce normalized probe counts, which were used in all downstream analysis. We defined copy number variable probes as those with either a coefficient of variation (CV) ≥0.1 among the 165 HapMap samples, or those that had counts ≥30% higher or lower than the population mean in ≥2 individuals.

### Derivation of Absolute Copy Numbers

Each Nanostring probe provides a count that is directly proportional to copy number of the target sequence in that individual. However, the relative counts given by different probes can vary depending on individual probe efficiencies. We therefore converted each probe count to absolute copy number using an independently calibrated data set. To derive absolute copy numbers in each individual, we compared our probe data with estimated copy number from whole-genome shotgun read depth analysis of individuals tested as part of the 1000 Genomes Project [51]. For each gene we extracted the corresponding copy number estimates from read depth data based on 85 individuals that were common to both studies. Where a gene or individual had more than one entry in read depth data we used the mean copy number. To ensure our approach was robust to noise in the individual estimates of copy number in read depth data, we calculated the median value for each Nanostring probe and the corresponding median copy number from read depth data in the 85 individuals tested. To convert Nanostring counts to absolute copy number, each NS count was divided by the corresponding median read depth value and multiplied with the corresponding median of the copy number estimate from the read depth data (as shown in the formula below), (Table S3).
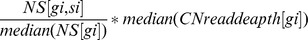
Where, gi: a NS probe i, si: a NS sample i, CNreaddepth: estimated copy number from read depth data

### Linkage Disequilibrium Analysis

To assess the degree of linkage disequilibrium (LD) between copy number of loci targeted by each probe and flanking SNP markers, we performed correlation analyses between probe counts and SNP genotypes. HapMap Phase II SNP genotypes for the 165 unrelated individuals studied (60 YRI, 60 CEU and 45 CHB) were downloaded (release 24, http://hapmap.ncbi.nlm.nih.gov), and SNPs with minor allele frequency (MAF) <0.1, Hardy Weinberg Equilibrium (HWE) p<0.05, or tri-allelic states were removed. To calculate LD between copy number for each of the 116 variable Nanostring probes and local SNPs, SNP genotypes were converted to integers (e.g. AA = 0, AB = 1 and BB = 2) and Pearson correlations with normalized probe counts performed to derive the coefficient of determination (R^2^). For each Nanostring probe, we extracted SNP genotype data within ±250 kb of each BLAT match of the probe sequence with ≥95% identity. Significance testing was performed using a permutation test in which the SNP genotypes and probe counts were randomly permuted across the samples in each dataset 10,000 times. For loci on the X and Y chromosomes correlations were calculated separately using male and female individuals. A median of 227 SNPs were tested per probe (range 8–1692) (Table S4). As SNPs that lie within copy number variable regions can often have erroneous genotypes as a result of underlying CNVs, we also performed more stringent filtering of SNPs, removing those that: (i) mapped by BLAT to >1 location in hg18 with 100% identity when considering a 51 bp interval centered on the SNP, (ii) overlapped with segmental duplications, or (iii) overlapped copy number variable regions identified by high-resolution oligonucleotide array [52] (Table S5).

### Gene Expression Analysis

We performed correlation analysis of Nanostring probe counts with steady-state mRNA levels of nearby transcripts. We used published Affymetrix exon array data available for 58 CEU and 59 YRI samples in our cohort [54]. For each probe we utilized all coordinates with ≥95% identity from BLAT analysis of each probe sequence, and extracted expression data for genes within ±500 kb of each probe match. Exonic expression measurements were summarized at the transcript level and transcripts that had mean log_2_ expression <6, or interquartile range <0.25 were removed. A median of 19 transcripts were tested for association with each probe (range 1–306). Pearson correlations were performed to derive the correlation coefficient (R) and coefficient of determination (R^2^) for each transcript:probe pair. Correlations for loci on the X and Y chromosomes were performed separately for males and females. Significance testing was performed by permutation in which the gene expression data and Nanostring probe counts were permuted across the samples in each dataset 10,000 times.

### DNA Methylation Analysis

We used published methylation data [57] from 133 HapMap unrelated samples (60 CEU and 73 YRI) typed with the Illumina Infinium HumanMethylation 450 BeadChip. We removed probes (i) mapping to multiple loci; (ii) with an overlapping SNP of MAF≥0.05 in the CEU or YRI 1000 genomes populations lying within 5 bp of the 3′ end of the probe, or at the targeted CpG; and (iii) probes mapping to the Y chromosome. Probes were then separated into those on autosomes (n = 461,272) and on chromosome X (n = 11,112). We performed color bias correction on red and green channels, background correction and quantile normalization using the lumi [79] and methylumi packages [80], as well as normalization to correct for bias between Infinium I and II probes using BMIQ [81]. Finally, after recalculating β-values from normalized intensities we set to missing those beta values with zero non-normalized intensity in both the methylated and unmethylated intensity channels or with detection p>0.01. Probes with >5% missing values were then removed. As a result, 448,817 autosomal probes were retained for the subsequent analyses in the 118 HapMap samples (60 CEU and 58 YRI) that overlapped with the set of samples included in our Nanostring study. For the 59 male samples (30 CEU and 29 YRI) for which we had copy number information, methylation data for probes located on the chromosome were processed separately to autosomal probes using the same methodology.

For each multicopy gene or macrosatellite we performed correlations with all methylation levels located within ±500 kb (excluding alignments on chromosome Y and chr_random scaffolds), resulting in a total of 98,941 methylation probes for which we measured the effect of copy number variation on the methylation patterns. We calculated Spearman's correlation (R) between Nanostring probe count and methylation levels (β-values), considering CEU and YRI populations as a single group as well as in either population separately (Table S7). In the analysis considering CEU and YRI as a single group, we corrected the significance of the correlations for multiple testing through permutations. To reduce the number of sites to correct for multiple testing and focus on the correlations more likely to be relevant we only retained the top 2% of methylation probes showing the greatest absolute Spearman's rho value (i.e. those with R>0.27 and R<−0.27), and then further filtered this set to retain only sites showing a β-value difference >0.1 between the lowest and highest observed copy number as predicted by the best linear fit (referred to as “normalized slope”). For permutation analysis we randomly selected from the initial 448,817 autosomal methylation probes 1,000 times and calculated the correlation between the estimated copy number values and the randomized β-values. The corrected p-value was calculated as the proportion of permutations that produced a correlation greater, in absolute terms, than the observed correlation value (two-tailed test). Further, we required Spearman's rho correlation to be also significant (p<0.05) in both CEU and YRI populations separately (Table **S**8). In order to avoid the confounder of X chromosome inactivation, we calculated correlations on chromosome X using male samples only using the combined CEU and YRI individuals in order to ensure sufficient sample size for a meaningful analysis (Table S9).

### Population Stratification of Multicopy Genes

We identified sites showing evidence of population stratification among the three HapMap populations tested using ANOVA-based F_ST_ statistics (ANOVA-F_ST_). Briefly, for each Nanostring probe we performed ANOVA across the European (CEU), African (YRI) and Asian (CHB) populations, and extracted the variance components from the ANOVA analysis, representing the between- and within-group variances. Variances were normalized within each population based on the total number of samples for that population. For loci on the X and Y chromosomes ANOVA-F_ST_ was calculated separately for female (F_ST female_) and male (F_ST male_) individuals, with the final F_ST_ reported being the mean of the F_ST male_ and F_ST female_.

### Selective Constraint of Multicopy Genes

To measure the extent of selective pressure on multicopy genes tested in our analysis during primate evolution, we analyzed the dN/dS ratios (ratio of the number of non-synonymous substitutions to the number of synonymous substitutions) of these genes in human versus chimpanzee, orangutan and macaque. High confidence one-to-one orthologs for all human Refseq genes and their dN/dS ratios were extracted from Ensembl v51 [60], and those with <80% amino-acid identity to human were excluded. A Kolmogorov-Smirnov test was applied to measure the difference in distributions of dN/dS ratios between multicopy genes and other orthologous Refseq genes.

### Comparative Analysis of Gene Copy Number in Human and Primates

Data for the five primate species tested were normalized and background corrected using the same method as in human. However, because probes were designed against the human reference sequence which is potentially divergent from the primate genomes, we first filtered the primate data to remove low-confidence data points that likely resulted from sequence divergence between human probe and primate target sequence. To avoid normalization artifacts, data normalization in each primate species was performed using only those invariant control probes whose count differed <20% from the relative mean signal for that probe in the 165 HapMap individuals (n = 10 in chimpanzee, 9 in bonobo, 7 in gorilla, 9 in gibbon, 7 in macaque). To ensure robust measurement of the relative copy number of each locus in primate versus human that was not influenced artifactually by poor probe binding, we retained data only for those probes exceeding a minimum identity threshold in each primate species. For loci showing a relative increase in copy number in a particular primate species versus human, we retained only those data points for which the probes had ≥95% sequence identity in that species. However, as mismatches between probe and target will generally result in reduced binding efficiency, we utilized a more stringent threshold of ≥98% sequence identity for any probes that showed a relative decrease in copy number in primate versus human. For CNVs on the sex chromosomes, relative copy number change in human versus primate was only based on individuals of the same gender.

### DNA Methylation and Quantitative PCR

DNA methylation levels were analyzed by MassARRAY EpiTYPER (Sequenom Inc.), using base-specific cleavage and Matrix-Assisted Laser Desorption/Ionization Time-of-Flight Mass Spectrometry (MALDI-TOF MS). Briefly, DNA samples isolated from HapMap lymphoblastoid cell lines (Coriell Institute, USA) were bisulfite converted using the EpiTect Bisulfite Kit (Qiagen) and amplified by PCR using promoter-tagged reverse primers and 10-mer sequence-tagged forward primers specific to each region (Table S11). Amplifications were verified using agarose gels, the PCR products transcribed *in vitro* using T7 polymerase, T-cleaved and processed using MALDI-TOF MS. DNA methylation levels were quantified using the EpiTYPER software.

For MSat10 copy number determination in a cell line with ChIP-seq data, we used a droplet digital PCR assay (QuantaLife, Bio-Rad Laboratories, Inc.). DNA from MS4221 and from 3 HapMap individuals with known MSat10 copy number were digested overnight at 37°C with *HaeIII* (New England Biolabs) to fragment the repeats. 20 ng of digested DNA was combined with QuantaLife Master Mix, PCR primers and VIC and FAM MGB labeled probes (Table S8). Droplets were generated from these mixes using the droplet generator (QuantaLife, Bio-Rad Laboratories, Inc.), PCR performed and the droplets assayed. Repeat copy number was calculated using QuantaSoft by comparing the relative fraction of positive droplets for the target and the reference amplicons.

### ChIP-Seq Analysis

ChIP was performed on lymphoblastoid cell line MS4221 [82] using polyclonal antibodies to H3K9me3, H4K20me3 and H3K4me2 (Abcam, Cambridge, MA; Catalog # ab8898, ab9053 and ab7766, respectively) as described previously [83], and libraries sequenced using an Illumina GAII instrument. Reads were aligned to hg18 using Bowtie with the following parameters: seed of 25 bp, maximum 2 mismatches, suppression (m) 20, and alignments (k) 20. After normalizing for total read number per sample, a 170 bp sliding window analysis (85 bp slide) was used to calculate the peak enrichment ratio between ChIP and input samples.

## Supporting Information

Figure S1 Independent Nanostring assays targeting polymorphic high copy number genes yield concordant results. Measurement of gene copy number using two independent probes targeted to different parts of the genes *TBC1D3*, *REXO1L1*, *PRR20A* and *CCL3*/*CCL4* all show high concordance, indicating that Nanostring probe counts provide reproducible measurements that are proportional to copy number over a wide dynamic range.
(TIF)

Figure S2 Comparison of copy numbers generated using Nanostring technology against other technologies for measuring high-copy number sequences. (a) Copy number estimates based on direct fragment sizing of both the *REXO1L1* tandem array by pulsed-field gel electrophoresis Southern blots show excellent correlations with Nanostring counts. (b) Comparison of Nanostring counts for the 8p23.1 *β-defensin* gene cluster with copy number measurements for this locus made using the paralog ratio test (PRT).
(TIF)

Figure S3 Comparison of copy numbers generated using Nanostring technology against those estimated by read depth analysis from whole genome shotgun sequencing. Each scatter plot shows normalized Nanostring counts for one probe versus copy number estimates of the same gene or macrosatellite locus made in the same individual by read depth analysis [51].
(TIF)

Figure S4 Loci showing highly divergent copy number between humans and other species of primate. 20 multicopy genes and macrosatellites showed ≥3-fold gain in copy number in at least one primate species versus the human mean measured by Nanostring assays. Each plot shows the relative fold-change versus the human mean. *PRAMEF14* shows the most extreme change detected, with an ∼18-fold increase in copy number in chimpanzee compared to the human mean, suggesting >200 copies of this gene in the chimpanzee individual tested. Note that as many probes do not have a perfect match in one or more of the other species tested, particularly in the more divergent species such as Gorilla, Gibbon and Macaque, many of these copy number expansions observed in different primates are almost certainly under-estimates.
(TIF)

Figure S5 Multicopy genes show evidence of altered selective pressures on amino acid sequence during recent primate evolution. Density plots showing the distribution of dN/dS ratios for multicopy genes (*green*) compared to all RefSeq genes (*red*) for human versus Orangutan and Macaque. There is a significant enrichment for elevated rates of non-synonymous substitution in multicopy genes versus the genome average in both species. This excess of non-synonymous amino-acid changes in recent primate evolution at multicopy genes is consistent with either reduced selective constraint and/or selection for proteins with altered function. Similar results are obtained when comparing human with chimpanzee (Figure 7).
(TIF)

Table S1 Description of Nanostring probe sequences used in this study and their mapping coordinates in hg18.
(XLSX)

Table S2 Nanostring counts for each probe in the 165 Hapmap individuals. Nanostring probe counts represent processed data after normalization and background correction of raw counts (See Materials and Methods). Abbreviations: CEU, CEPH Europeans from Utah; YRI, Yoruba from Ibadan Nigeria; CHB, Han Chinese in Beijing.
(XLSX)

Table S3 Summarized absolute copy numbers of multicopy genes and macrosatellites in 165 HapMap individuals. Abbreviations: INV: Invariant, VAR: Variant.
(XLSX)

Table S4 Results of linkage disequilibrium analysis between probe counts and all SNPs located within ±250 kb of each probe position. For each probe we report the SNP identified with the highest R^2^ value with probe count within ±250 kb. Abbreviations: CEU, CEPH Europeans from Utah; YRI, Yoruba from Ibadan Nigeria; CHB, Han Chinese in Beijing, ALL: Includes CEU, YRI, CHB individuals combined.
(XLSX)

Table S5 Filtered results of linkage disequilibrium analysis between probe counts and SNPs within ±250 kb of each probe position. This shows similar results to those presented in Table S4, except for each probe we report the SNP identified with the highest R^2^ value with probe count within ±250 kb after removing SNPs that: (i) overlap regions of common copy number variation (CNVs with ≥10% MAF, [52]), (ii) overlap annotated segmental duplications, or (iii) the 51 bp sequence centered on the SNP does not map uniquely within the genome using BLAT. Abbreviations: CEU, CEPH Europeans from Utah; YRI, Yoruba from Ibadan Nigeria; CHB, Han Chinese in Beijing, ALL: Includes CEU, YRI, CHB individuals combined.
(XLSX)

Table S6 Results of correlation analysis between probe counts and expression level of genes within ±500 kb of each probe BLAT position. Copy number of eight multicopy genes (*CCR5*, *FCGR*, *GSTM*, *GSTT2*, *KRTAP1-1*, *MAFG*, *MAGEA9*, *UGT2B15*) shows significant correlation (p<0.01) with their own expression level. Abbreviations: CEU, CEPH Europeans from Utah; YRI, Yoruba from Ibadan Nigeria, All: CEU, YRI and CHB individuals combined; R and R^2^ are derived from Pearson correlations.
(XLSX)

Table S7 Results of correlation analysis between probe counts and methylation level of autosomal CpGs within ±500 kb of each probe BLAT position. Reported in this table are all pairwise correlations between Nanostring counts and CpG methylation levels (β-values) with unadjusted p<0.01 observed in either the CEU+YRI populations combined as a single group, or in any of the two populations separately (autosomal loci only). Abbreviations: CEU, CEPH Europeans from Utah; YRI, Yoruba from Ibadan Nigeria.
(XLSX)

Table S8 Filtered results of correlation analysis between probe counts and methylation level of autosomal CpGs within ±500 kb of each probe BLAT position. Reported in this table are pairwise correlations between Nanostring counts and CpG methylation levels (β-values) that show permutation p<0.01 in the combined CEU and YRI populations and which also validate (unadjusted p<0.01) in each in each population separately (autosomal loci only). In addition the effect size on methylation, as measured by the absolute normalized slope, must be >0.1. Abbreviations: CEU, CEPH Europeans from Utah; YRI, Yoruba from Ibadan Nigeria.
(XLSX)

Table S9 Filtered results of correlation analysis between probe counts and methylation level of X-linked CpGs within ±500 kb of each probe BLAT position. Reported in this table are pairwise correlations between Nanostring counts and CpG methylation levels (β-values) that show permutation p<0.01 in the combined CEU and YRI male populations (chromosome X probes only). In addition the effect size on methylation, as measured by the absolute normalized slope, must be >0.1. Abbreviations: CEU, CEPH Europeans from Utah; YRI, Yoruba from Ibadan Nigeria.
(XLSX)

Table S10 Probe alignments, probe counts and relative copy numbers obtained in five species of primate compared to the human mean.
(XLSX)

Table S11 Description of primers used for Sequenom MassARRAY EpiTYPER and QuantaLife droplet digital PCR.
(XLSX)
