# Supplementary figures and images for: Digital Genotyping of Macrosatellites and Multicopy Genes Reveals Novel Biological Functions Associated with Copy Number Variation of Large Tandem Repeats
Source: PLoS Genet. 2014 Jun 19;10(6):e1004418. doi: 10.1371/journal.pgen.1004418 (PMC4063668; doi:10.1371/journal.pgen.1004418)

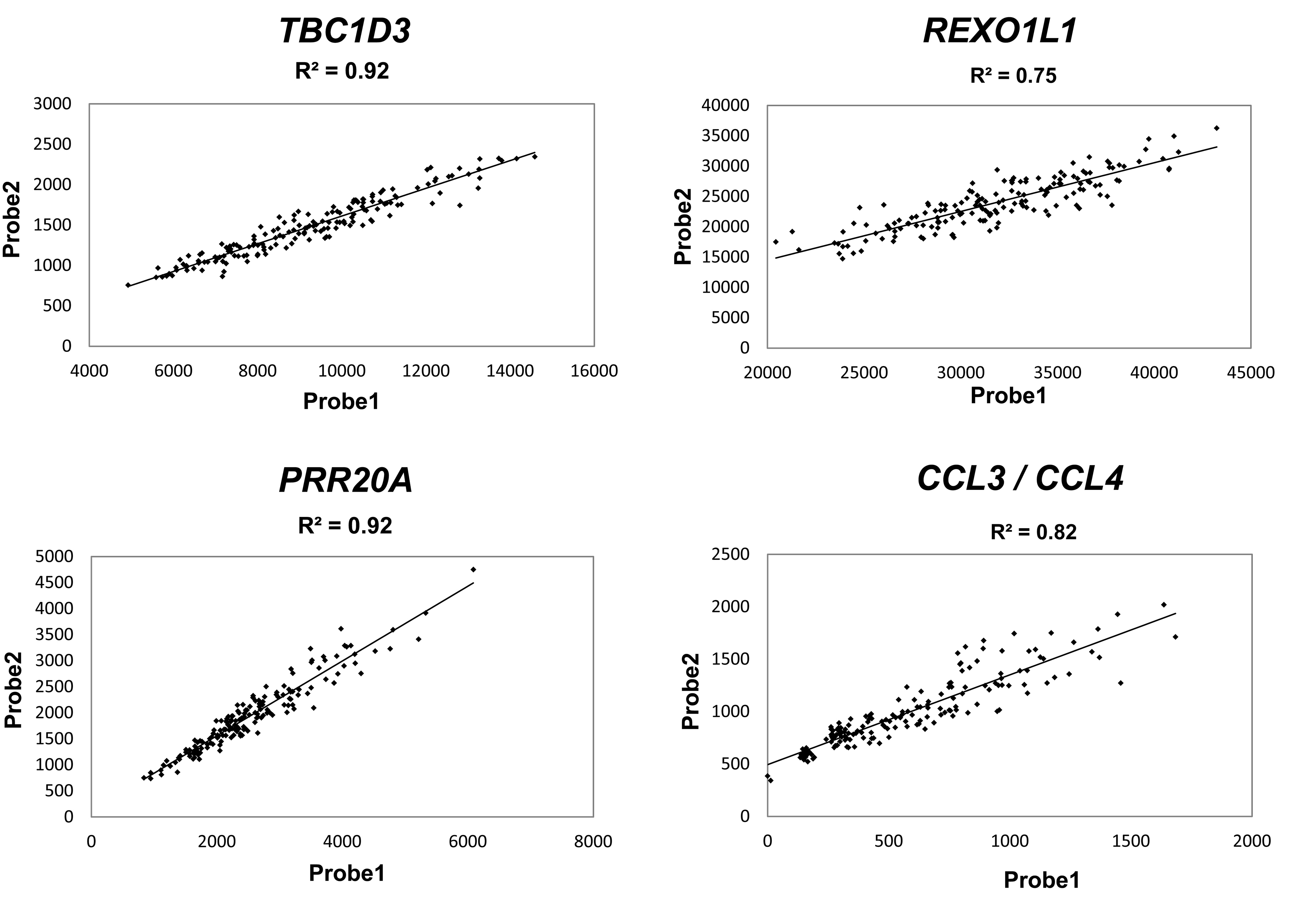

Supplement: Figure S1 — Independent Nanostring assays targeting polymorphic high copy number genes yield concordant results. Measurement of gene copy number using two independent probes targeted to different parts of the genes TBC1D3, REXO1L1, PRR20A and CCL3/CCL4 all show high concordance, indicating that Nanostring probe counts provide reproducible measurements that are proportional to copy number over a wide dynamic range. (TIF) [file pgen.1004418.s001.tif]

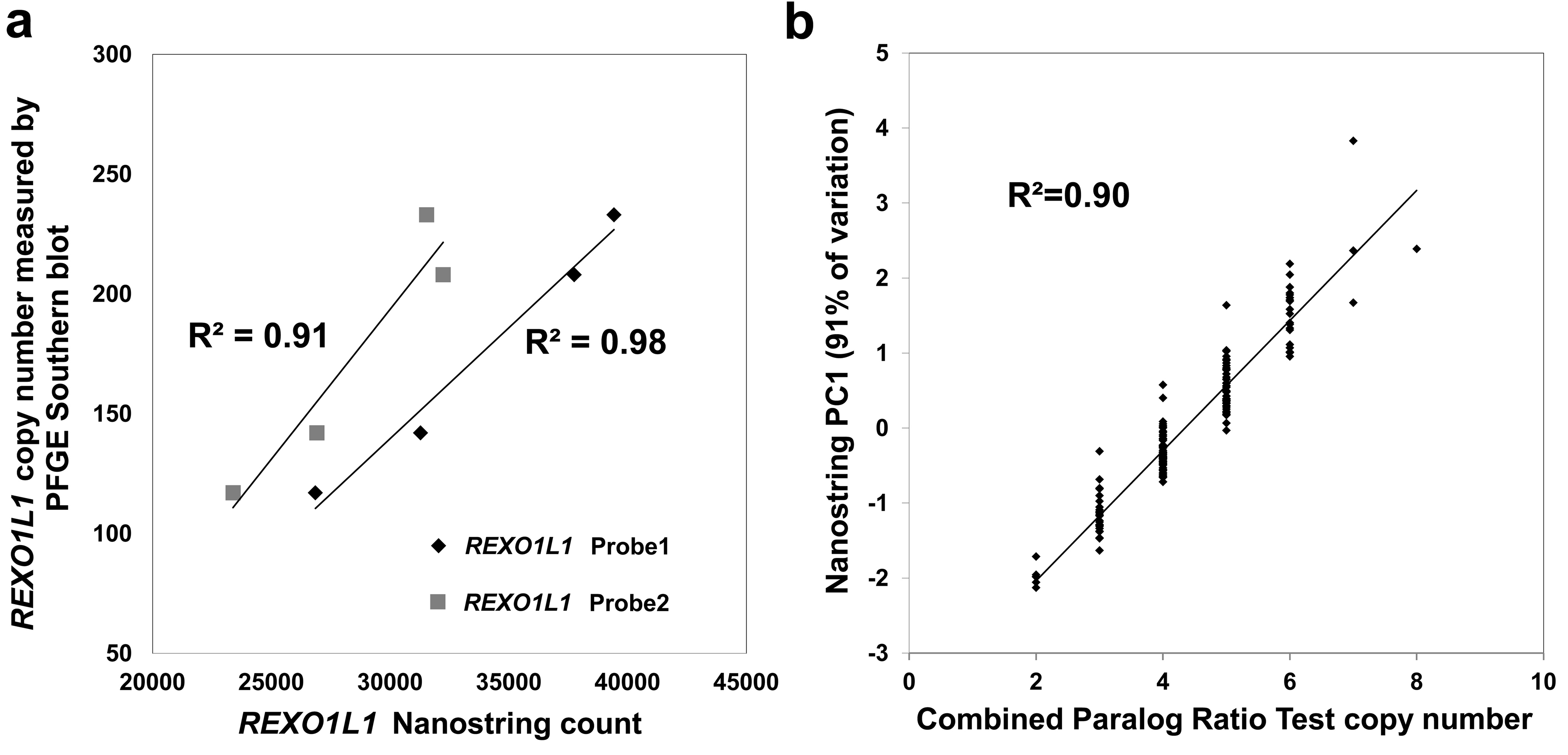

Supplement: Figure S2 — Comparison of copy numbers generated using Nanostring technology against other technologies for measuring high-copy number sequences. (a) Copy number estimates based on direct fragment sizing of both the REXO1L1 tandem array by pulsed-field gel electrophoresis Southern blots show excellent correlations with Nanostring counts. (b) Comparison of Nanostring counts for the 8p23.1 β-defensin gene cluster with copy number measurements for this locus made using the paralog ratio test (PRT). (TIF) [file pgen.1004418.s002.tif]

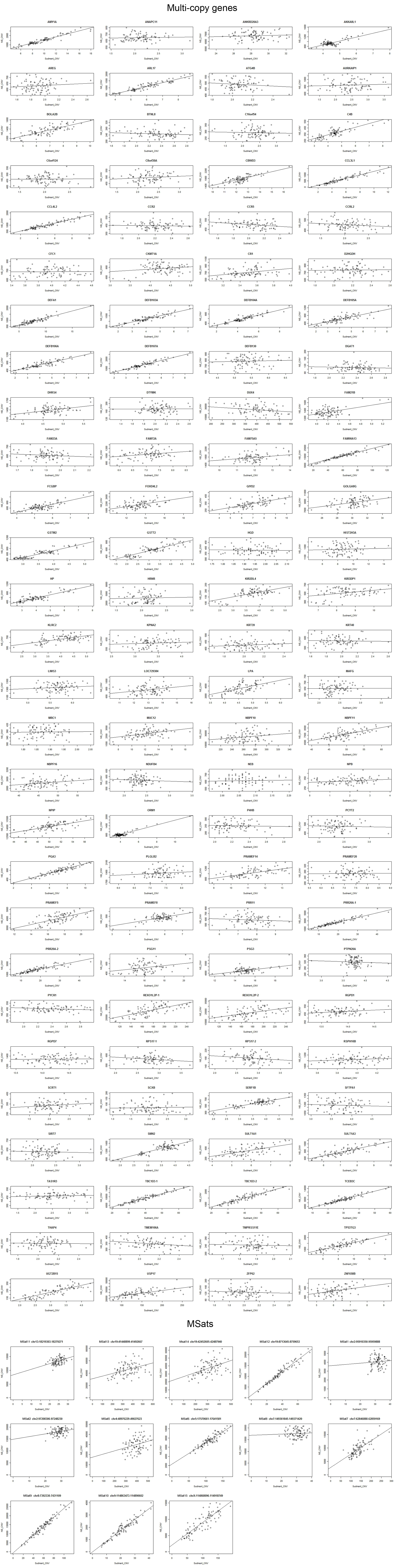

Supplement: Figure S3 — Comparison of copy numbers generated using Nanostring technology against those estimated by read depth analysis from whole genome shotgun sequencing. Each scatter plot shows normalized Nanostring counts for one probe versus copy number estimates of the same gene or macrosatellite locus made in the same individual by read depth analysis [51]. (TIF) [file pgen.1004418.s003.tif]

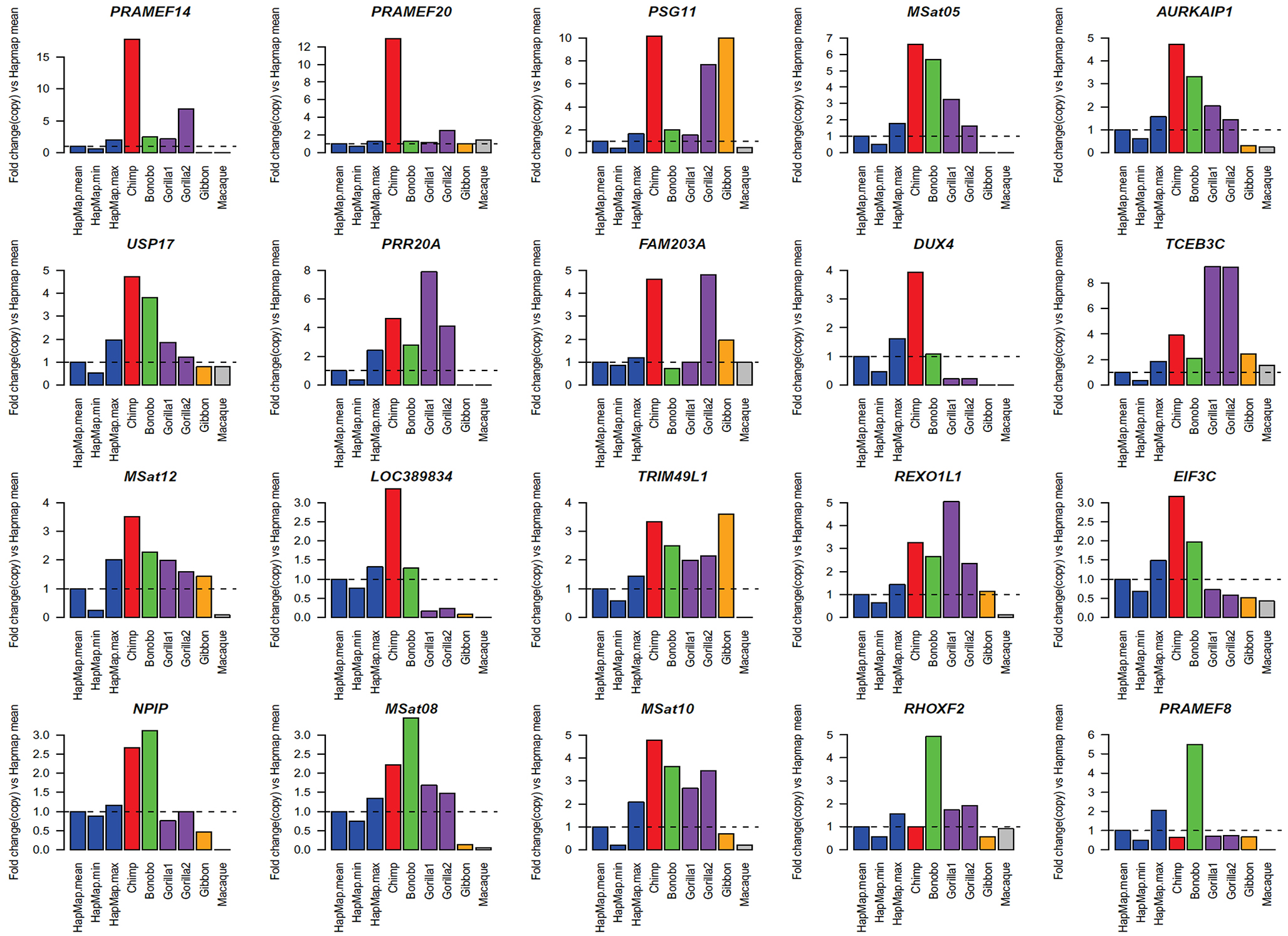

Supplement: Figure S4 — Loci showing highly divergent copy number between humans and other species of primate. 20 multicopy genes and macrosatellites showed ≥3-fold gain in copy number in at least one primate species versus the human mean measured by Nanostring assays. Each plot shows the relative fold-change versus the human mean. PRAMEF14 shows the most extreme change detected, with an ∼18-fold increase in copy number in chimpanzee compared to the human mean, suggesting >200 copies of this gene in the chimpanzee individual tested. Note that as many probes do not have a perfect match in one or more of the other species tested, particularly in the more divergent species such as Gorilla, Gibbon and Macaque, many of these copy number expansions observed in different primates are almost certainly under-estimates. (TIF) [file pgen.1004418.s004.tif]

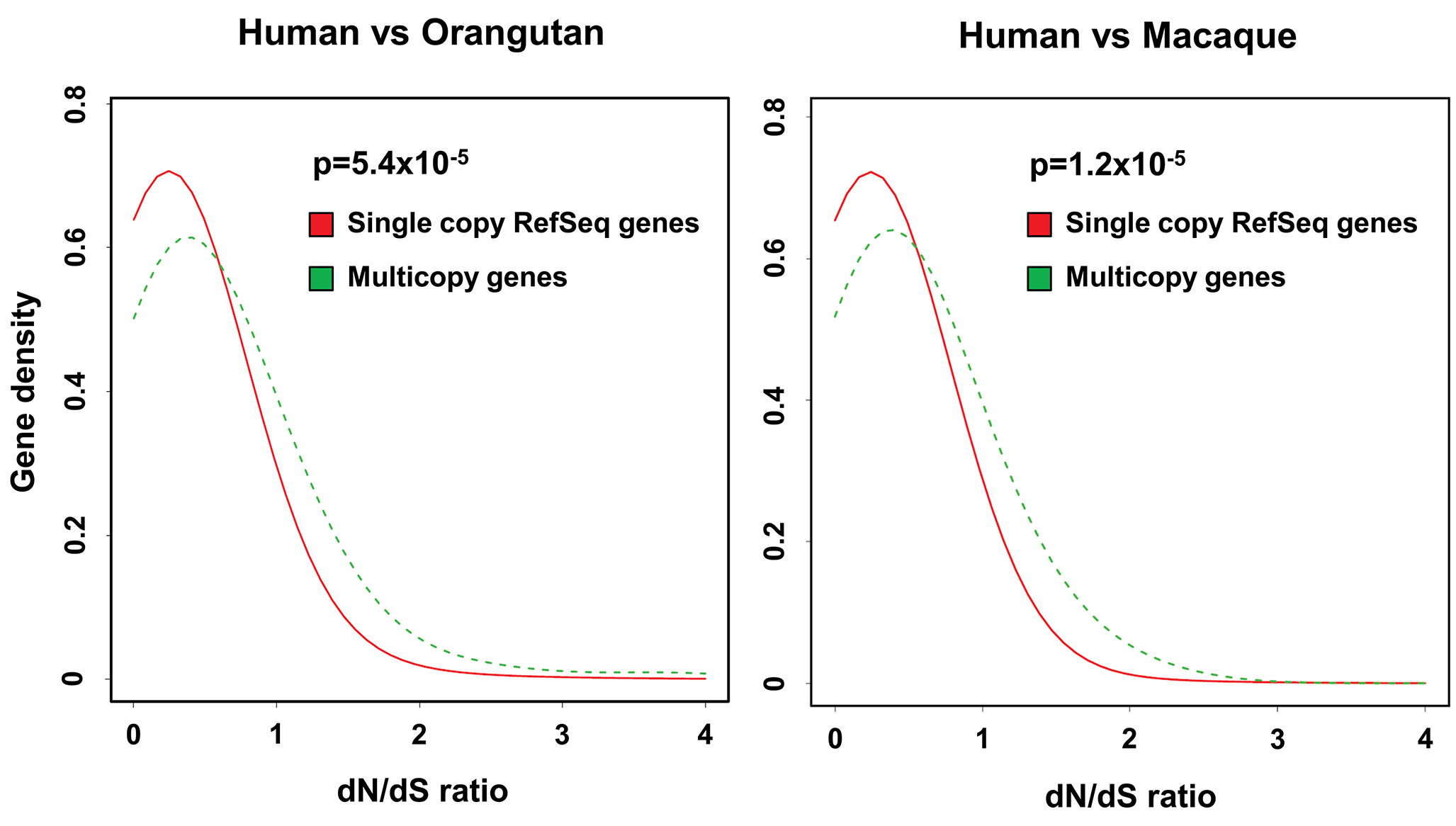

Supplement: Figure S5 — Multicopy genes show evidence of altered selective pressures on amino acid sequence during recent primate evolution. Density plots showing the distribution of dN/dS ratios for multicopy genes (green) compared to all RefSeq genes (red) for human versus Orangutan and Macaque. There is a significant enrichment for elevated rates of non-synonymous substitution in multicopy genes versus the genome average in both species. This excess of non-synonymous amino-acid changes in recent primate evolution at multicopy genes is consistent with either reduced selective constraint and/or selection for proteins with altered function. Similar results are obtained when comparing human with chimpanzee (Figure 7). (TIF) [file pgen.1004418.s005.tif]
